# Novel chitosan/diclofenac coatings on medical grade stainless steel for hip replacement applications

**DOI:** 10.1038/srep26653

**Published:** 2016-05-24

**Authors:** Matjaž Finšgar, Amra Perva Uzunalić, Janja Stergar, Lidija Gradišnik, Uroš Maver

**Affiliations:** 1 University of Maribor, Faculty of Chemistry and Chemical Engineering, Smetanova ulica 17, SI-2000 Maribor, Slovenia; 2 University of Maribor, Faculty of Medicine, Institute of Biomedical Sciences, Taborska ulica 8, SI-2000 Maribor, Slovenia

## Abstract

Corrosion resistance, biocompatibility, improved osteointegration, as well the prevention of inflammation and pain are the most desired characteristics of hip replacement implants. In this study we introduce a novel multi-layered coating on AISI 316LVM stainless steel that shows promise with regard to all mentioned characteristics. The coating is prepared from alternating layers of the biocompatible polysaccharide chitosan and the non-steroid anti-inflammatory drug (NSAID), diclofenac. Electrochemical methods were employed to characterize the corrosion behavior of coated and uncoated samples in physiological solution. It is shown that these coatings improve corrosion resistance. It was also found that these coatings release the incorporated drug in controlled, multi-mechanism manner. Adding additional layers on top of the as-prepared samples, has potential for further tailoring of the release profile and increasing the drug dose. Biocompatibility was proven on human-derived osteoblasts in several experiments. Only viable cells were found on the sample surface after incubation of the samples with the same cell line. This novel coating could prove important for prolongation of the application potential of steel-based hip replacements, which are these days often replaced by more expensive ceramic or other metal alloys.

The demographic pyramid in the western world shows a rather steep increase in increasingly older people^1^. Regardless of the effectiveness of healthy ageing strategies, it is expected that many people will still experience significant functional decline in old age. Frailty, connected with osteoporosis and the consequential bone damage, is highly prevalent in old age and constitutes a major public health problem^2^,^3^. Hip replacement is among the most common orthopedic procedures in the patient group above 60 years of age, with an average annual increase of over 25% between 2000 and 2009^2^.

As reducing costs in public health services is of vast significance, stainless steel is widely and commonly used for manufacturing dental, orthopedic, and other implantable biomedical devices^4^,^5^. Although medical-grade stainless steel possesses several favorable characteristics, such as ease of fabrication, mechanical properties, corrosion resistance, and relatively low cost, there is still much room for improvement in terms of its bioactivity, especially in relation to possible active pharmacotherapy. Other commonly used materials in orthopaedic clinical practice include cobalt and titanium based alloys, as well as several non-metal materials, e.g. polymethylmethacrylate (PMMA) ceramis etc^6^,^7^,^8^,^9^.

Based on these facts, the focus of this study is the development of novel coatings on AISI 316LVM stainless steel (ASTM 138, DIN 1.4441) and proof of their applicability in a physiological environment. Therefore, the main goal is to develop advanced steel-based artificial hips. Herein, we will be focused on the corrosion susceptibility of coated AISI 316LVM stainless steel compared with uncoated material to check if these coatings have any influence on the matrix material. The composition of the coating (CHI/DCF-based, CHI – chitosan, DCF – diclofenac) was carefully chosen in order to enable controlled drug delivery into surrounding tissues, as well as for being completely biocompatible or even promoting bone cell proliferation (osteointegration). CHI is a naturally occurring polysaccharide that has been used in numerous biomedical applications, such as wound dressings^10^,^11^, hemostatic materials^12^,^13^, drug/gene delivery depots^14^,^15^,^16^ and tissue engineering scaffolds^17^,^18^. Different CHI-based materials were also successfully used in bone tissue engineering applications^19^,^20^,^21^. Some research studies report on CHI use as a coating either alone or in combination with other components in settings, where corrosion inhibition was required^22^,^23^. Several studies have also proven the potential of CHI to be used either as part or as pure source of mechanically stable bone tissue engineering scaffolds^19^,^20^,^21^. Finally, CHI was previously shown to even possess antimicrobial properties, which is also desired in orthopedic surgery^24^,^25^,^26^. DCF was chosen due to the double beneficial effect of NSAID drugs in relation to hip replacement, namely their activity in reducing the pain experienced by the patient after surgery, and their anti-inflammatory activity in decreasing the body’s possible immune response due to the implant. Drugs inducing both of the mentioned activities are commonly administered in orthopedic applications and are usually applied as systemic therapy. Advanced and improved steel-based artificial hips can prolong the life cycle of such hips and consequently decrease the probability of repeated hip replacement surgical procedures, often connected with inappropriate artificial hips^27^,^28^,^29^. Such novel solutions can in turn significantly decrease the overall treatment costs per patient^30^,^31^,^32^. Amongst the flood of hydroxyapatite-based coatings on medical-grade stainless steel^33^,^34^,^35^, our composition represents a novel approach to biocompatible and bioactive coatings on materials for orthopedic applications.

AISI 316LVM stainless steel has a strictly defined composition with a low carbon content, and is primarily used in biomedical applications. Currently its most important applications are in manufacturing implants, such as femoral heads, hip stems, and bone screws^36^,^37^. Such implants are significantly less expensive than those made of ceramics, polymers, composites, and alloys^32^,^38^,^39^. Its low cost combined with superior mechanical properties, possible biofunctionality and biocompatibility make it one of the most commonly used materials in orthopedics still today^6^,^40^,^41^. One of the advantages of metal implants over ceramics is that they are not brittle. If the implant cracks in the human body this can lead to the release of particles of different sizes, and can result in serious inflammation of the surrounding tissues, accompanied by severe pain or even patient death^42^,^43^,^44^. On the other hand, the possible corrosion of metal implants in the constantly changing body fluids of complex composition still represents an important issue that needs to be carefully controlled. Corrosion-induced ion release is potentially toxic, possibly leading to serious health implications (i.e. the inactivation of enzymes, protein coagulation, damage to nucleic acids, and even cancer)^45^,^46^,^47^,^48^. In order to prevent corrosion of metallic implants, surface modification of stainless steel is likely to improve steel-body compatibility, and hence prevent its corrosion after prolonged exposure to demanding conditions present in the body. Various modifications have already been tested to improve the corrosion resistance characteristics of AISI 316LVM stainless steel for biomedical applications, e.g. mechanical polishing^49^, chemical functionalization, etc^50^,^51^,^52^.

Although the biocompatibility of AISI 316LVM stainless steel has already been proven, bioactive and biocompatible coatings on artificial hip prostheses can improve the interaction potential with exposed bone tissue either by attracting cells or by active therapeutic action through controlled (prolonged) release of incorporated drugs. Coatings that, in addition to the mentioned characteristics, simultaneously reduce corrosion and prevent particle release into the surrounding tissue are desired. However, the latter is not of primary concern as AISI 316LVM is a highly resistive material by itself. The main purpose of the coating presented herein is therefore not to improve the metal implant corrosion performance (the best possible scenario would be that these coatings would improve the corrosion resistance of the whole system), but to provide novel functionalities to the hip implant, in terms of the implant’s controlled therapeutic action (incorporation and delivery of drugs) and/or the promotion of osteointegration (measured as biocompatibility and possible cell proliferation).

Non-steroid anti-inflammatory drugs (NSAIDs) are important drugs in relieving pain, fighting fever, and decreasing inflammation. Based on scientific and clinical evidence, pain can significantly slow down the healing process (mostly through stress-induced release of hormones such as cortisol and norepinephrine), which results in decreased patient quality of life as well as in exponentially increased personal and public expenditures^53^,^54^,^55^. The same can be said for inflammation, which is a common complication related to any kind of implant, hip replacement being no exemption^56^,^57^. Mostly NSAIDs are taken through systemic administration (i.e. in the form of pills), whereas several approaches have been and are being researched with regard to their integration into different formulations for local therapy^58^,^59^,^60^,^61^.

In this study, tests were carried out in 0.9 wt.% NaCl, and at 37 °C in order to simulate physiological solution and body temperature. At this stage of investigation, a NaCl solution instead of simulated body fluids (SBF) was selected as the occurring phenomena can be more accurately determined in simpler medium, which is important for preliminary studies related to novel materials (as in our case). Moreover, NaCl is the most corrosive component of SBF and therefore corrosion study does not suffer due to the selection of simpler medium. The novelty in our approach is not only in the combination of a biocompatible polysaccharide (CHI) and an NSAID (DCF) drug as a coating for steel-based hip replacement materials, but even more in providing possible local and localized drug delivery for improved material acceptance in orthopedic applications. Additionally, our tests of the as-prepared samples were conducted in an environment similar to the actual application. Furthermore, the coatings were prepared considering the final material biocompatibility with bone-derived cells as well as its capability to deliver incorporated drugs in a controlled manner. Both characteristics are important for possible orthopedic applications. Using a simple coating strategy and combining as few components as possible for its preparation could allow for interesting future scale-up options. *In vitro* release performance was tested on a Franz diffusion cell drug dissolution system, while the biocompatibility testing was based on a primary cell culture derived from human bone. Proven biocompatibility on such a cell line is a very good indication of possible promoted osteointegration. Last but not least, our approach can be used as a testing platform for other prosthetics in small laboratory batches, potentially significantly decreasing their overall development costs.

## Experimental

### Sample and solution preparations

Physiological solution with 0.9 wt.% NaCl was prepared with ultra-pure water with a resistivity of 18.2 MΩ cm. NaCl (pro analysis) was purchased from Carlo Erba, Italy and low molecular weight CHI (high purity: CHI ≥ 93%) and DCF (analytical grade, purity ≥ 98.5%) from Sigma Aldrich, USA. AISI 316LVM was purchased from Tiger International (Shanghai, China). The composition is specified in Table 1. Using this material, samples were cut out in the shape of discs 15 mm in diameter. The AISI 316LVM samples were ground using a rotating device under a stream of water using 320- and 500-grit SiC paper (provided by Struers, Ballerup, Denmark). Samples were ground in one direction until all imperfections were removed and the surface was covered with a uniform pattern of scratches. The grinding direction was changed four times by turning the sample through 90° to minimize abrasion. Finally, the samples were cleaned ultrasonically in a bath of 50% ethanol/50% ultra-pure water (by volume) and afterwards thoroughly rinsed with ultra-pure water^62^. For this and all following experiments, ultra-pure water (18.2 MΩ cm at 25 °C) produced by the Milli-Q® system (EMD Millipore Corporation, USA) was used.

The as-prepared AISI 316LVM samples were further used as substrates for film preparation using a spin coater (SPIN-150i-NPP, SPS Vertriebs GmbH, Germany). For this purpose, the CHI and DCF solutions were prepared separately as follows. 1 g of CHI was dissolved in 80 mL ultra-pure water. The pH of the solution was initially adjusted to 3 with glacial acetic acid (Sigma Aldrich, Germany) and constantly stirred until complete dissolution. Either diluted NaOH or HCl (from Sigma Aldrich, France) were then used to adjust the pH to 5.5. The total volume of the solution was adjusted to 100 mL, prior to the last preparation step, which was to fix the ionic strength of the solution to 150 mM with NaCl. DCF solution was prepared by mixing 20 mg of DCF with 20 mL ultra-pure water (to obtain a concentration of 1 mg/mL). The layer-by-layer coating preparation using spin-coating was performed as follows. The AISI 316LVM substrate was placed on the spin coater. A drop of the as-prepared CHI solution (100 μL) was placed on the substrate and spin-coated with a spinning speed of 2500 rpm and an acceleration of 1000 rpm/s for 180 s. A similar procedure was used for the second layer by placing 100 μl DCF solution on the substrate with the first coated layer. Following the same protocol (using CHI and DCF solutions), the procedure was repeated until multi-layer coatings on the AISI 316LVM substrate were formed. The preparation was concluded with drying under nitrogen of high purity (99.999 wt.%) and stored in a desiccator until further used. The prepared samples were named according to the number of respective layers, namely 3CHI3DCF-coated and 3CHI2DCF-coated, where the number represents the number of CHI and DCF layers.

### Sample characterization

Coating characterization includes attenuated total reflectance-infrared (ATR-IR) measurements and the evaluation of sample topography, morphology, roughness and homogeneity using profilometry and atomic force microscopy (AFM) as follows.

ATR-IR spectra were recorded using an Agilent Cary 630 FTIR spectrometer with the diamond ATR module at a scan range of 4000–650 cm^−1^. The scans were performed on three different places in 8 repetitions on each sample surface after preparation of each respective layer of both samples.

For surface analysis of the coated and uncoated AISI 316LVM samples a profilometer, model Form Talysurf Series 2 (Taylor-Hobson Ltd.), was employed. The instrument has a vertical resolution of about 5 nm. The measurement starts in one direction on the surface and the 3D-topography is obtained by combining several measurements in parallel directions. The measured data were processed with TalyMap Gold 4.1 software to calculate the mean surface roughness and to create a surface profile. To level the profile, corrections were made to exclude general geometrical shape and possible measurement-induced misfits^63^.

The surface morphology and roughness parameters of the as-prepared samples for profilometry measurements were characterized by atomic force microscopy (AFM) in tapping mode with an Agilent 5500 AFM multimode scanning probe microscope (Agilent, Santa Barbara, CA). The images were acquired after drying the samples in a stream of dry high-grade (99.999 wt.%) nitrogen. The images were scanned using silicon cantilevers (ATEC-NC-20, Nanosensors, Germany) with a resonance frequency of 210–490 kHz and a force constant of 12–110 N m^−1^. All measurements were performed at room temperature. For all samples images of 5 × 5 μm^2^ were recorded with a resolution of 2048 × 2048 pixels^64^. All images were processed and the corresponding roughness calculated using WSxM software^65^.

### Electrochemistry

Electrochemical measurements were carried out at 37 °C using the same equipment as described before^63^. The prepared AISI 316LVM discs as described above were employed as a working electrode.

Electrochemical impedance spectroscopy (EIS) experiments were performed in the frequency range from 100 kHz to 5 mHz with 10 points per decade and a 10 mV (peak to peak) amplitude of the excitation signal at the open circuit potential, *E*_ocp_. The EIS signal was fitted with Gamry’s EChem Analyst software, version 6.25. Cyclic polarization measurements were performed after 10 h of immersion using a potential sweep rate (*ν*) of 0.1 mV/s. Measurements started at an initial potential (*E*_i_) of –0.25 V vs. the open circuit potential, *E*_ocp_, and continued with increasing potential in the anodic direction until the signal reached a current response of 1 mA cm^−2^. Then the potential sweep was reversed in the cathodic direction until the repassivation potential was reached.

Several replicate measurements for every electrochemical experiment were performed. Outliers in the EIS measurements and fitting procedure were discarded by the Grubbs statistical test^66^. On the other hand, in the case of cyclic polarization measurements, a representative curve is given^63^.

### *In vitro* drug release testing

*In vitro* drug release studies were performed using an Automated Transdermal Diffusion Cells Sampling System (Logan System 912–6, Somerset, USA). The drug-loaded samples 3DCF2CHI and 3CHI3DCF were slowly placed heads up into the Franz diffusion cell. The receptor compartment was filled with phosphate buffered saline – PBS (Sigma-Aldrich, Germany, PBS is composed of 0.01 M phosphate buffer, 0.0027 M potassium chloride, and 0.137 M sodium chloride, pH 7.4 at 25 °C) and its temperature was maintained at 37 °C. During the dissolution testing the medium was stirred continuously with a magnetic bar at 50 rpm, placed below the Franz diffusion cell. Samples were collected over a period of 24 h at different time intervals (1 min, 5 min, 10 min, 20 min, 30 min, 60 min, 120 min, 180 min, 240 min, 300 min, 360 in, and 1440 min), while the released/dissolved DCF concentration in the receptor medium was determined using UV-Vis spectrophotometry (Cary 60 UV-Visible Spectrophotometer, Agilent, Germany) by quantification of the absorption band at 276 nm. The withdrawn sample volumes were replaced by fresh PBS with a stable temperature of 37 °C. Due to sample withdrawal, followed by sample dilution through media replacement, sink conditions were ensured. In the calculation of concentrations using the Beer-Lambert law, this dilution was accounted for. All release studies were performed in three parallels. For determination of the incorporated DCF amount, samples 3CHI2DCF-coated and 3CHI3DCF-coated were placed into a glass beaker, filled with 15 mL of PBS and tightly sealed. UV/Vis spectrophotometry was used to determine the amount of drug released on a daily basis until the concentration no longer changed.

### Primary cell origin and culture

A human backbone specimen was obtained from one male patient after spine surgery. The included patient was informed and the specimen was taken after obtaining written consent. Prior to surgery, no systemic disease or any treatment was reported for the included patient. The study was conducted in accordance with the *Declaration of Helsinki* and its subsequent amendments, and further approved by the Ethics Committee of the University Medical Centre Maribor (Maribor, Slovenia). Primary osteoblasts were isolated as previously described^67^. Cells were cultured in Dulbecco’s modified Eagle’s medium (DMEM, Gibco, Grand Island, NY, USA) supplemented with 10 wt.% fetal bovine serum (FBS, Gibco, Grand Island, NY, USA), 100 U/mL streptomycin, and penicillin. Only cells between passage 2 and 4 were used in the study.

### Biocompatibility testing

Two types of biocompatibility testing were performed as follows. Initially, biocompatibility testing with the as-prepared primary osteoblast cell culture was performed using solutions of 3CHI3DCF-coated and 3CHI2DCF-coated samples, withdrawn at the desired time intervals during the release testing. For this purpose, these solutions were incubated with the osteoblast cell culture in P96 microtitre plates. Each well was filled with cell suspension containing 60,000 cells and after 24 hours, when the cells attached, it was supplemented with the dissolved 3CHI3DCF-coated and 3CHI2DCF-coated sample solutions, as described above. The solutions used were diluted in a ratio of 1:2 in the cell culturing media (Advanced Dulbecco’s modified Eagle’s medium (ADMEM, Gibco, Grand Island, NY, USA)), supplemented with 5 wt.% FBS. Cytotoxic effects on the cell culture were observed after 24 hours of incubation at 37 °C and 5 wt.% CO_2_. Cell viability was determined via the reduction reaction of the tetrazolium salt MTT (3(4,5-dimethylthiazolyl-2)-2,5-diphenyltetrazolium bromide).

The second test was performed directly on the as-prepared samples using the primary osteoblast cell culture, isolated as described above. Prior to testing all samples were sterilized under UV light for 30 minutes. Coated AISI 316LVM stainless steel substrates (samples 3CHI3DCF-coated and 3CHI2DCF-coated) were incubated with the osteoblast cell culture in P24 microtitre plates. For the control, cover slide of the same size was used. Each well was filled with cell suspension containing 50,000 cells and the ADMEM media, supplemented with 5 wt.% FBS. After 72 h of incubation the cell reached confluence, the MTT assay was performed as described below.

### Cytotoxicity by MTT reduction assay

The reduction of tetrazolium salts is now widely accepted as a reliable way to examine cell proliferation. The yellow MTT is reduced by metabolically active cells, in part by the action of dehydrogenase enzymes, to generate reducing equivalents, such as NADH and NADPH. The resulting intracellular purple formazan can be solubilized and quantified by spectrophotometric means. The MTT cell proliferation assay measures the cell proliferation rate and conversely, when metabolic events lead to apoptosis or necrosis, the reduction in cell viability. The MTT reagent yields low background absorbance values in the absence of cells. For each cell type the linear relationship between cell number and the signal produced is established, thus allowing an accurate quantification of changes in the rate of cell proliferation^68^,^69^. An MTT cell proliferation assay was performed according to Mosmann^70^. The test protocol used was somewhat different for the respective sample types (withdrawn solutions and on substrates).

For the withdrawn sample solutions, the as-prepared osteoblast cells (10,000 cells/well) were seeded into 96-well microtitre plate with a final volume of 100 μL of DMEM medium supplemented with 10 wt.% FBS. Cells were treated with the withdrawn samples during the *in vitro* release testing for up to 360 min. These samples were then incubated for 24 h at 37 °C in a humidified atmosphere containing 5 wt.% CO_2_. After treatment, cell proliferation was determined using the standard reduction of the tetrazolium salt MTT^68^,^69^.

For the substrate based samples, all samples (including control) were moved after 72 h to a fresh P24 microtitre plates. These were filled with 1 mL fresh media (ADMEM + 5 wt.% FBS) and 200 μL of MTT. After 3 h of incubation at 37 °C in a humidified atmosphere containing 5 wt.% CO_2_, the media was removed and MTT assay was performed using the standard reduction of the tetrazolium salt MTT^68^,^69^.

### Live/Dead kit and confocal microscopy

The viability of the primary osteoblast cell culture on 3CHI2DCF-coated and 3CHI3DCF-coated samples was assessed using the Live/Dead assay (Sigma-Aldrich, Germany). For this purpose samples were prepared in the same way as for the MTT assay on substrates. After 96 h of incubation, all tested samples were rinsed with PBS, incubated in 4 mM calcein AM and 2 mM ethidium homodimer in PBS for 30 minutes at 37 °C, and finally washed with PBS. Subsequently, the constructs were observed under a confocal microscope (SP5 AOBS, Leica Microsystems, Germany). Viable cells are detected by the presence of intracellular esterase activity, which converts calcein-AM to calcein and produces a green fluorescence. Dead cells are recognized by ethidium homodimer, which enters the cell through the damaged plasma membrane and binds with deoxyribonucleic acid to produce red fluorescence. The observation using confocal microscopy was performed on three different surface regions on each sample.

## Results and Discussion

### Sample preparation and coating characterization

The aim of this study was to produce a novel biocompatible and bioactive multi-layer coating on AISI 316LVM stainless steel that would not promote or that it would even inhibit corrosion of the stainless steel base and promote osteoblast growth. As shown hereinafter, all mentioned characteristics can be achieved by using a combination of a known biocompatible polymer, chitosan (CHI), and a non-steroid anti-inflammatory drug (NSAID), diclofenac (DCF). The multi-layered structure was chosen, since a higher amount of layers ensured that enough material to induce therapeutic action could be deposited on the AISI 316LVM stainless steel surface, and it also enabled the control and prolonged release of DCF. Controlled release is important to prevent possible local drug overdoses that could result in localized tissue damage. Prolonged release on the other hand, plays an important role in terms of the applicability of the developed system, namely to ensure release for a time frame of one day, after which systemic therapy can be employed effectively and safely.

**Figure 1:**
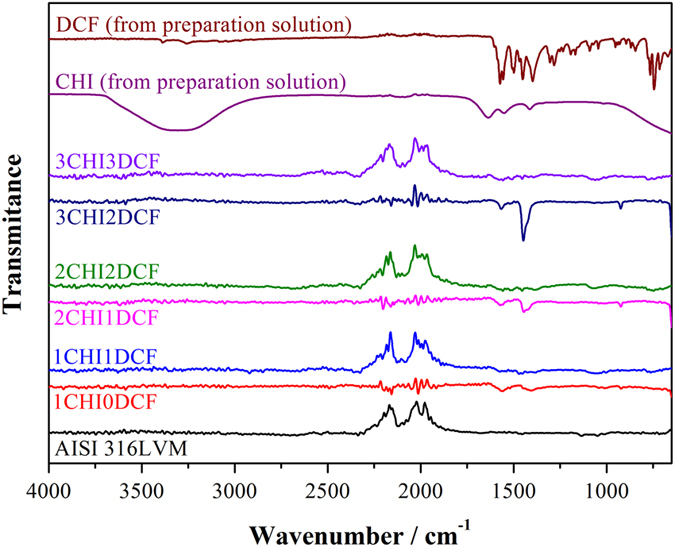
ATR – IR spectra taken after the alternating preparation of CHI and DCF layers. Included are spectra of pure CHI and DCF as well as for bare AISI 316LVM substrate. The name above respective spectra follows the chosen sample names, where the number before CHI and DCF represents the number of respective layers.

The multi-layered nature of the developed coatings was evaluated using an ATR-IR analysis after preparation of each respective layer (Fig. 1). To characterize separate layers, spectra of pure CHI and DCF are also included (using a droplet of respective ultra-pure water solutions of CHI and DCF).

A broad peak between 3700–3000 cm^−1^ that corresponds to O–H vibration is observed for the pure CHI sample spectra, while the latter is not evident for the respective layers, where CHI is the upper layer. The reason for this might be H-bonding between water and CHI. Three peaks that correspond to CHI, can be observed in all spectra with CHI on top. These include the CH scissoring between 1422–1380 cm^−1^, the asymmetric stretching vibrations of the –NH group at 1650–1510 cm^−1^, and the C=O stretching vibrations at around 1654 cm^−1 ^71. After adding the second and third CHI layer (spectra for 2CHI1DCF and 3CHI2DCF) another peak becomes visible, namely the C–O–C stretching vibrations at around 900 cm^−1^. Due to addition of DCF layers the emergence of several new peaks can be observed. Peaks that can be assigned to C–Cl stretching vibrations are visible in the region of 650–780 cm^−1^, while a band corresponding to HC–N–CH bending vibration is observed at 1376 cm^−1^. At 1577 cm^−1^, R=C=O stretching vibration is observed. Additional peaks corresponding to R–C=O stretching and CH_2_ bending are visible at 1305 and 1462 cm^−1 ^61,72. Although small in intensity, these peaks are visible for all samples with DCF as the top layer.

Considering the alternative manner of the appearance of respective peaks that correspond either to CHI or DCF, the proposed sample structure can be confirmed.

**Figure 2:**
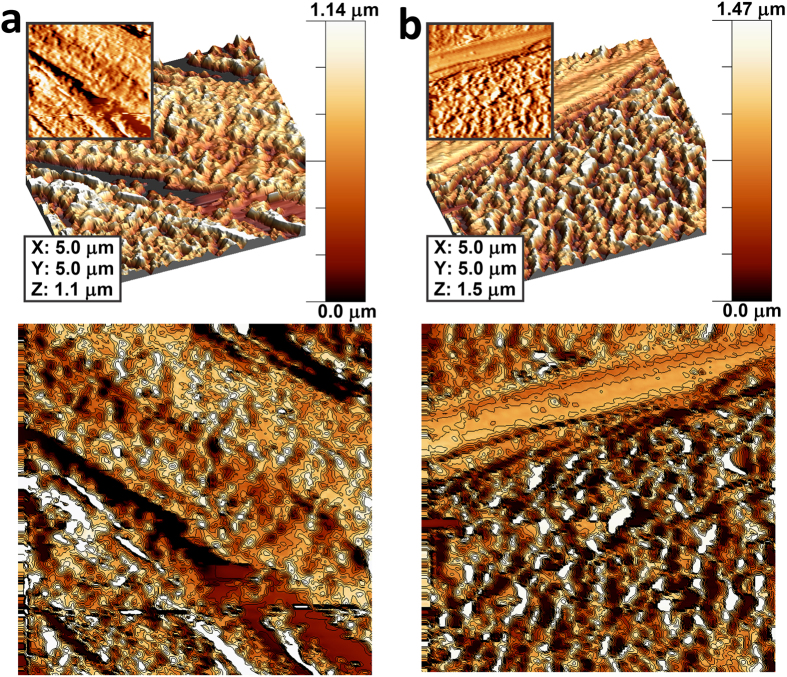
AFM images of the 3CHI2DCF-coated sample (**a**) and 3CHI3DCF-coated sample (**b**). The samples’ surfaces are shown in 3D and 2D (inlays), as well as with added contour diagrams to expose the different grain size due to different final layers.

In order to analyse the surface morphologies and the small scale homogeneity of the two coated samples, where the final layer was either CHI (3CHI3DCF-coated) or DCF (3CHI2DCF-coated), AFM measurements were performed. The results are presented in Fig. 2 in the form of three images for the respective samples to show their most important features in 2D, 3D, and as a contour image. Upon first glance at the 2D and 3D images, it might seem that there are not any significant differences between the samples, but the contour images expose different surface features more clearly. The most evident difference is in the apparent grain size of the particulate and mountain-chain-like surface features, which can be attributed to the different chemical properties of the respective final layers, as well as to the fact that during the spin-coating process the media evaporates at different rates^73^, influencing the respective sample morphologies. For the 3CHI2DCF-coated sample (Fig. 2a), the surface structures formed seem smaller and form thinner surface connections. The 3CHI3DCF-coated sample, on the other hand, shows bigger features with thicker, more interconnected surface structures (Fig. 2b). An additional finding based on the AFM analysis is that the samples, regardless of the mentioned surface features, exhibit surface holes, most likely pores, with a very similar diameter (these are ion-conducting paths, as described above with EIS measurements). The latter is an important finding in agreement with the *in vitro* release results, explained below. Overall, the AFM analysis showed homogenously distributed surface features on respective samples on the measured scale. These findings are also in agreement with the results obtained through profilometry as described below.

**Figure 3:**
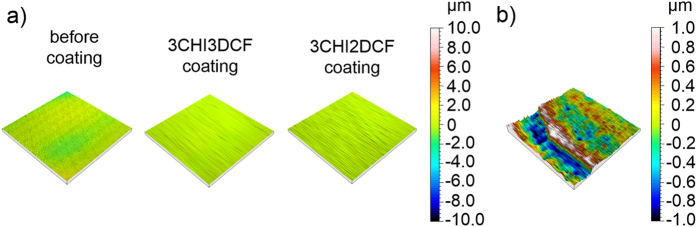
(**a**) 3D-profiles of the uncoated AISI 316LVM stainless steel substrate (left), 3CHI3DCF-coated (middle) and 3CHI2DCF-coated (right) samples after their preparation and (**b**) 3D-profile of the coating edge. In both cases, a centred height scale is shown on the right side of the figure.

Surface profiles were obtained by employing a stylus profilometer and a 1 mm^2^ spot size (Fig. 3). The results showed a very high homogeneity of surface profiles for both samples (Fig. 3a), demonstrating homogenous coatings on a larger scale. For comparison, the surface profile of the AISI 316LVM stainless steel substrate is added. Although the AFM results (the measurements were performed on a much smaller surface area) exhibit a rougher surface profile, the overall surface roughness (based on profilometry measurements), seems very low (the average *S*_a_ values – 3 replicas – determined with stylus profilometer measurements for the 3CHI3DCF-coated and 3CHI2DCF-coated samples are 0.118 μm and 0.126 μm, respectively). This is an important indication of good control over the preparation procedure, which is, together with homogenous coatings, a much desired property in medical applications. Finally, by coating the AISI 316LVM stainless steel substrate, the average *S*_a_ value was significantly decreased (for the uncoated substrate the determined *S*_a_ value was 0.385 μm).

Profilometry measurements were additionally used for determination of respective layer thickness. Since determination of the latter by other means is far from straightforward, using profilometry for this purpose seemed the best option. The layer thickness was determined on the edges (a stair) of the coating on the AISI 316LVM stainless steel substrate. Respective layers were determined to be approximately 1.5 μm thick as shown in Fig. 3b.

### Electrochemical measurements

#### Electrochemical impedance measurements

**Figure 4:**
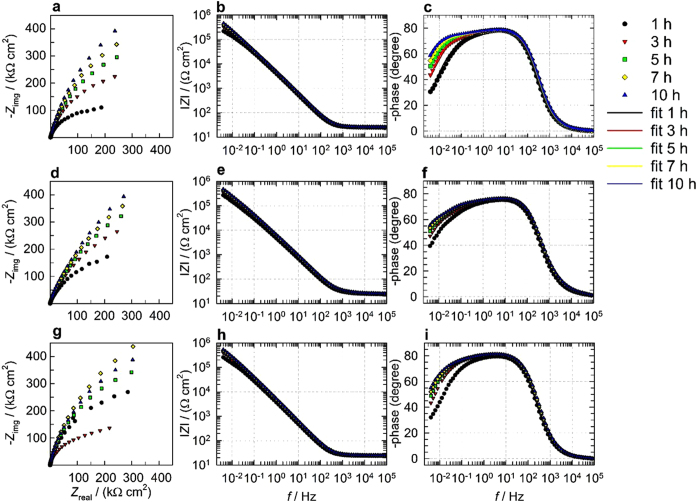
Electrochemical impedance measurements for (**a–c**) uncoated, (**d–f**) 3CHI3DCF-coated, and (**g–i**) or 3CHI2DCF-coated AISI 316LVM stainless steel after 1, 3, 5, 7, and 10 h of immersion in physiological solution, (**a**) Nyquist and (**b,c**) Bode plots. Figure c also shows fitted EIS data using the model in Fig. 5.

Figure 4 shows EIS measurements for uncoated and both coated AISI 316LVM in physiological solution after different immersion periods at 37 °C. In Fig. 4b,e,h a horizontal asymptote is observed in the high *f* region at 1–100 kHz (*f* stands for frequency). Moreover, in the highest measured *f* region, the phase angle approaches 0° (Fig. 4c,f,i). The latter two observations are characteristic of resistive behavior and correspond to the response of the uncompensated resistance *R*_Ω_, as explained below. In the middle *f* region (around 0.1–100 Hz), the slope of the Bode plot in Fig. 4b,e,h is close to 1 and the phase angle tends toward −90° in Fig. 4c,f,i, indicating capacitive behavior. In the lowest measured *f* region (below 0.1 Hz), the |*Z*| from the initial stage of immersion (1 h) constantly increases up to 10 h of immersion, as seen in Fig. 4b,e,h. The increase in resistance vs. time is also noted in Fig. 4a,d,g (the loop is increasing). Moreover, in the lowest measured *f* region no horizontal asymptote was developed (Fig. 4b,e,h), which indicates that a diffusion process is involved in the corrosion process. This assumption can also be supported by the fact that the phase angle approaches 45° with decreasing *f* in Fig. 4c,f,i. Therefore, the diffusion process will be taken into account in the EIS fitting procedure explained below^74^.

**Figure 5:**
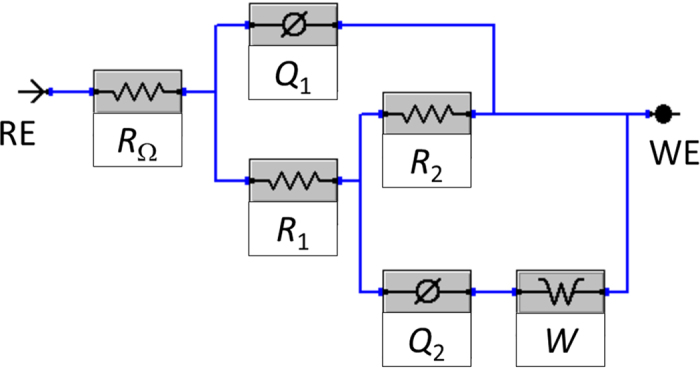
The EEC model used to fit the EIS response.

In order to fit the EIS measured data, the nested *R*_Ω_(*Q*_1_(*R*_1_(*Q*_2_(*R*_2_*W*))) equivalent electrical circuit (EEC) model given in Fig. 5 was employed. *Q* represents a non-ideal capacitance and is only used for the fitting procedure^75^. Based on *Q*-values, double-layer, oxide, or surface layer capacitances *C* were calculated as *C*_x_ = (*R*_x_*Q*_x_)*n*_x_/*R*_x_^63^,^76^ and are reported in Table 2. As seen in Fig. 4c, the measured and fitted data match well, which confirms the appropriateness of the fitting procedure.

The first relaxation process, *R*_1_*Q*_1_, describes the surface layer characteristics. In the case of the sample without coating, *R*_1_*Q*_1_ describes the resistance *R*_1_ and the capacitive behavior *Q*_1_ of the oxide surface layer (as this is the passive layer on the stainless steel). The resistance *R*_1_ characterizes the resistance of the surface layer, which comprises pockets filled with an electrolyte solution that are ion-conducting paths, where the electrolyte solution is very different than the bulk solution outside of this surface layer. Therefore the *R*_1_ value is significantly higher than *R*_Ω_ (the surface layer also restricts ion movement, which significantly contributes to higher *R*). On the other hand, for the samples with coatings, *R*_1_ and *Q*_1_ describe the combined properties of the coating and the oxide layer.

Next, a second relaxation process, *R*_2_*Q*_2_, describes the charge transfer resistance (*R*_2_) and double-layer capacitance (represented by *Q*_2_). Moreover, the diffusion process was also taken into account, as already predicted above (designated as the Warburg element *W* in Fig. 5).

The model in Fig. 5 and the parameters in Table 2 therefore explain the corrosion process, the surface layer characteristics, and the diffusion process. Therefore coated and uncoated AISI 316LVM stainless steel is under mixed kinetic-controlled and diffusion-controlled process in physiological solution at 37 °C.

**Figure 6:**
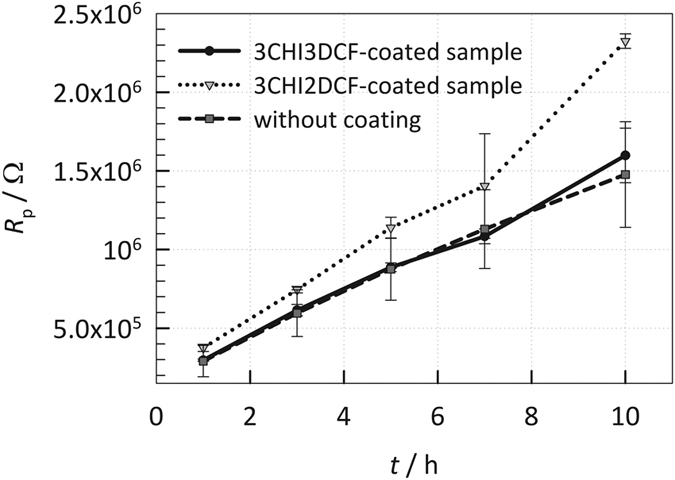
*R*_p_ fitted EIS values by the EEC model in Fig. 4 and the corresponding 95% confidence interval for AISI 316LVM with (DCF or CH on top) or without coating, measured after 1, 3, 5, 7, and 10 h of immersion in physiological solution at 37 °C. Higher *R*_p_ represents higher general corrosion resistance.

Based on fitted EIS data in Table 2 *R*_p_ (polarization resistance) values were calculated for different immersion periods as *R*_p_ = *R*_1_ + *R*_2_ and are given in Fig. 6. The confidence interval was determined as 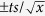, where *t* is a Student’s *t*-distribution, *s* is the standard deviation, and *x* is the number of measurements. *R*_p_ is a measure of how the metal resists against transferring the electron to the electroactive species in solution. Therefore, the higher *R*_p_ is, the more resistive the sample is against general corrosion. There is no significant difference in *R*_p_ values for the sample with DCF coating on top (3CHI3DCF-coated sample) and AISI 316LVM without coating up to 7 h of immersion. However, after 10 h of immersion the sample with DCF coating is more resistive. On the other hand, in the case of CHI coating on top (3CHI2DCF-coated sample), *R*_p_ values are higher for all measured immersion periods compared with the other two samples.

Therefore, the CHI coating on top (the 3CHI2DCF-coated sample) represents the sample most resistive to general corrosion. For all three cases, the *R*_p_ values increase with increased immersion time, indicating that the passive layer (most likely oxide) is developing and contributing to a more resistive sample and the CHI coating (3CHI2DCF-coated sample) reinforces that oxide layer.

#### Cyclic polarization measurements

Cyclic polarization measurements were performed after 10 h of immersion for the three samples. A relatively slow sweep rate was employed (0.1 mV/s) is order to avoid capacitive contribution due to the potential change so that every step resembles the kinetic or diffusion process of the system, as projected above with EIS measurements.

**Figure 7:**
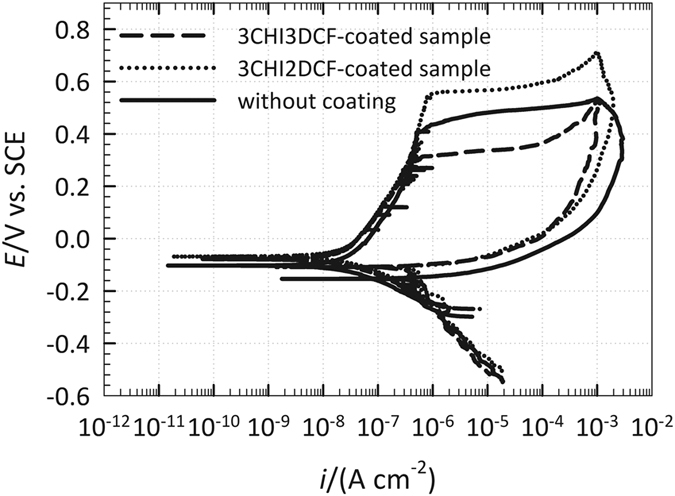
Cyclic polarisation measurements for AISI 316LVM with coatings DCF coating (the 3CHI3DCF-coated sample) or CHI coating (the 3CHI2DCF-coated sample)) or without coating, measured after 10 h of immersion in physiological solution.

The breakdown potential (*E*_bd_) is the potential where the current suddenly increases in the forward scan. The open circuit potential (*E*_ocp_) is the potential on the forward scan at which the cathodic current becomes zero. The repassivation potential (*E*_rp_) is the potential on the reverse scan at which the anodic current becomes zero. Therefore, the latter two potentials are defined where the current changes polarity (anodic vs. cathodic current). *E*_bd_, *E*_ocp_, *E*_rp_, *E*_sw_, and *E*_i_ potentials and scan direction (short dash arrows) are designated in Fig. 7. In order to extract certain properties of these samples, the differences between the particular potentials, *E*_bd_–*E*_ocp_, *E*_rp_–*E*_ocp_, *E*_bd_–*E*_rp_, were used^77^.

At potentials more positive than *E*_bd_, the metal or alloy loses its passivity and pits or crevices start to develop. The sudden increase of current density (*i*) in that potential region may also correspond to transpassive oxidation of metal species, such as chromium and nickel. By reversing the potential sweep in the cathodic direction at *E*_sw_, pit or crevice propagation does not cease, however it carries on at a lower rate until *E*_rp_ is reached. At that potential, pits or crevices repassivate and cease to propagate, however they remain on the surface^78^,^79^. In general, the higher the potential difference *E*_bd_–*E*_ocp_ is, the slower the localized initiation rate that occurs^79^,^80^, therefore, the slowest localized initiation rate would occur for the CHI-coated sample (the 3CHI2DCF-coated sample), followed by uncoated and 3CHI3DCF-coated samples (Fig. 7). For all three samples, the current density in the reversed direction is higher than in the forward direction (a current hysteresis), suggesting a lack of resistance to localized corrosion after its initiation (however, this does not necessarily mean that this material will suffer from localized corrosion – crevice and pit formation).

The extent of crevice corrosion resistance is related to the potential difference of *E*_bd_–*E*_rp_, where a lower difference represents more resistant metallic material^81^. *E*_rp_ values are close for the 3CHI2DCF-coated and 3CHI3DCF-coated samples, whereas the *E*_rp_ of the uncoated sample is at more negative potentials. The potential difference *E*_bd_–*E*_rp_ is the lowest for the 3CHI3DCF-coated sample, followed by the uncoated and 3CHI2DCF-coated samples, indicating that the extent of crevice corrosion damage would follow the same trend, and therefore is the lowest for the 3CHI3DCF-coated sample. The potential difference *E*_rp_–*E*_ocp_ signifies how the localized corrosion damage can repassivate after its formation. However, as the *E*_rp_ is always at more negative potentials than *E*_ocp_, this potential difference is negative for all three samples, indicating that localized corrosion damage (if formed, as here oxidation was forced by applied potential) cannot be repaired (repassivated)^82^,^83^.

### *In vitro* drug release studies

**Figure 8:**
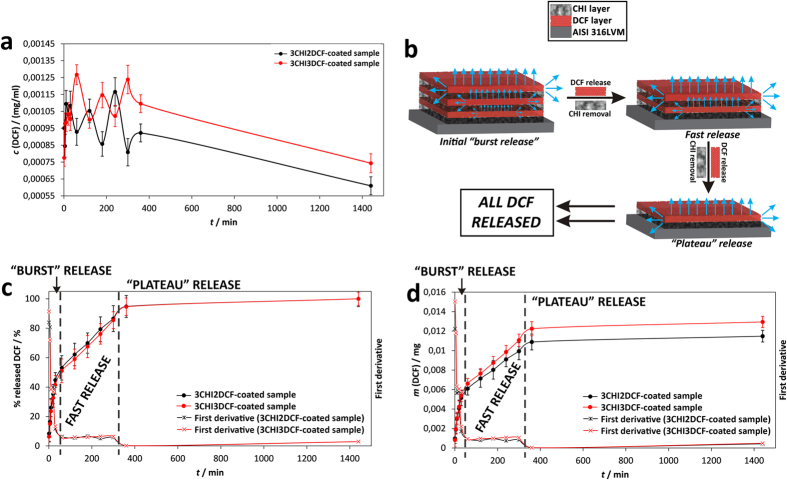
The results of *in vitro* drug release testing with the corresponding 95% confidence intervals: (**a**) DCF concentration as a function of time, (**b**) shows a schematic depiction of the proposed release scenario in light of the obtained results and the calculation of first derivatives, (**c**) the cumulative mass of released DCF as a function of time, and (**d**) the % of released DCF as a function of time. The shown mass in (**c**) represents the mass in 1 ml of the withdrawn sample from 15 ml of dissolution media. In order to calculate the overall released mass, therefore, a factor of 15 of needs to be accounted for. First derivatives, calculated from cumulative DCF released mass and the % of released DCF, were added to (**c**) and (**d**) respectively.

Since our initial aim was to prepare a simple multi-functional drug-based coating on AISI 316LVM stainless steel, drug release studies were among the most important features of our samples. Three important findings are presented in the three respective diagrams describing our results, as shown in Fig. 8, and are explained in the following subchapters. For all curves shown in Fig. 8 the confidence interval was determined as described above, namely as 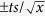, where *t* is a Student’s *t*-distribution, *s* is the standard deviation, and *x* is the number of measurements.

#### The concentration of released DCF as a function of time

Figure 8a shows the changing concentration as a function of release time during the *in vitro* release testing. The initial 360 minutes of release are marked by a pulsatile release profile. Such a profile is in agreement with the sample composition, where the drug is deposited on the AISI 316LVM stainless steel surface in alternating layers of CHI and DCF (as schematically depicted in Fig. 8b). The number of respective peaks/valleys corresponds to the number of drug-related layers in the sample. The most likely scenario is that DCF is released from the layer in direct contact with the media in a “burst” like fashion. Smaller amounts of DCF are also released from the DCF layers below (as shown in Fig. 8b), contributing to the overall very fast release during this part of the curve. After the mentioned time frame, the DCF layer in direct contact with the media is dissolved, the exposed CHI layer, which until then acts as a barrier for the DCF layer below, gets peeled off, and the bottom DCF layer is dissolved, and so on. Therefore, the mechanism of DCF dissolution is presumably for the most part governed by the concentration gradient between the material surface and the media (and hence diffusion) process from the surface to the bulk solution. A smaller contribution to the overall dissolution process is due to the lower DCF layers, where DCF has to either penetrate through all of the above layers or is released sideways from the coatings. The mentioned scenario is schematically shown in Fig. 8b.

#### The cumulative mass of released DCF as a function of time

Figure 8c presents the release results from the perspective of the cumulative amount of released DCF. Namely, during dissolution testing the withdrawn samples (comprised of 1 mL media with the, until then, dissolved amount of DCF) were replaced by fresh media. Figure 8c therefore shows DCF masses as a function of time, where this dilution was already accounted for. Two important results can be deducted from such a representation. The first is that by adding another DCF layer on top of the as-prepared samples (3CHI3DCF-coated), the amount of released DCF is affected only after the initial 30 minutes of release. The importance of this finding is that if such a material were to be used clinically, patients would experience the same initial pain reduction irrespective of the dose, while the final DCF dose would still be in accordance with the patient’s physical characteristics (as seen from the decline in the release profile after the initial 30 minutes). It is also in agreement with our scenario involving the “peeling-off” of CHI layers, as mentioned above. The second important finding evident in Fig. 8c is that by adding another DCF layer on top of the last CHI layer, the corresponding amount of DCF released can be increased without a significant effect on the overall release kinetics (the difference between the two presented curves is in the inclination of the curve between 30 to 360 minutes of release). Hence, the proposed preparation process allows us to fine-tune the final dose of DCF by a very simple method. The therapy is similarly efficient in lowering pain in the initial 30 minutes, whereas the final dose, which is connected with the individual patient, can be fine-tuned without significant effect on the overall release, hence pain and inflammation will be lowered with the same clinical efficiency for the respective patients.

#### The amount of released DCF as a function of time

Finally, Fig. 8d shows the results in the form of the percentage of DCF released as a function of time. This percentage was calculated by using the final release amount (as determined by exposing the sample to the media for a prolonged time and evaluating the concentration changes on a daily basis until the concentration stabilized). The endpoint was determined to be after 1 day, therefore the release results are shown for this period of time. The most important finding from Fig. 8d complements the second one, namely that not only can the dose be increased (even adjusted) by simply adding an additional DCF layer, but that although the incorporated DCF amount was increased, all incorporated DCF in the respective samples was released in almost the same time frames (as seen in the very similar release profiles in Fig. 8d). This finding is very important, if we consider potential orthopedic application, where in accordance with the patients’ physical characteristics (mostly mass and overall health assessment), the dose of a drug is adjusted. Moreover, during orthopedic surgical procedures, which require anesthesia, it is usually desired that patients wake up as soon as possible, and this should happen regardless of the dose. To put our system into this perspective, we should mention that DCF provides pain-reducing and anti-inflammatory activities. After surgery it is of course expected that patients experience pain after waking up, which can be managed by our system. The second DCF activity, namely the anti-inflammatory effect, can be provided by our system in a localized manner, namely around the replaced hip. The latter could ensure better uptake of the implant by lowering the potential immune system response due to the implanted artificial hip. Since most patients should wake up soon after surgery (also because prolonged unnecessary anesthesia has been connected with serious implications for patient health)^84^,^85^,^86^, the time frame of one day, which is the time period in which the DCF is released from our samples, is ideal for both mentioned effects. After a period of one day, effective systemic therapy can be optimally adjusted with efficiency confirmation by the (then) awake patient. Systemic therapy with regard to pain reduction is always employed after orthopedic surgery, while immunomodulatory and/or anti-inflammatory drugs to lower the possibility of implant rejection by the body are administered if necessary. Last but not least, it was previously proven that CHI provides antimicrobial activity^87^,^88^. The latter could additionally lower the possibility of complications related to the hip replacement procedure, since our approach would prevent initial infection during the procedure, whereas consecutive infections, following surgery, are always handled systemically due to the possible occurrence of antimicrobial resistance with locally applied antibiotics^89^.

### Evaluation of the release mechanism

Our objective was to understand the release from our coating systems also from the mechanistic perspective. For this purpose, we applied several models to describe the respective release profiles according to^90^,^91^,^92^. The authors of the present study have studied different materials and their release performances before^58^,^93^,^94^,^95^,^96^,^97^. Unfortunately, as simple as our system may seem (schematically depicted in Fig. 8b), the available models were not able to describe our profiles with the desired statistical weight. One possibility is to present the profiles as a three-section release mechanism (as can be deduced from the release results in the form of concentration – Fig. 8a). The latter can be confirmed by calculating the first derivatives of the release profiles as shown in Fig. 8a. Such representation proved that the release mechanism of our samples is complex, and most likely several phenomena add to the final release profile (see the discussion above), as three distinct sections can be deduced from the first derivatives of the results shown in Fig. 8a. The latter also confirms why none of the available models can efficiently describe our results, and why the discussion above in relation to Fig. 8a probably describes the actual scenario. Considering the above discussion, the three release regions could be an initial (very fast) burst release, a second (still) fast release, and a final release part, where the remainder of the DCF is released and a plateau is reached (Fig. 8a,b).

Finally, as mentioned above in the discussion of the AFM measurement results, the surface morphology, although perhaps different for both samples on the nanoscale, exhibits very similar holes on the surface, which most likely represent pores. Similar morphologies are in agreement with the very similar release profiles obtained for both samples, since the interaction of the sample surface with the media probably proceeds in a similar fashion.

### Consideration of possible clinical applicability

To proceed even further in consideration of possible clinical application of the proposed coating system on AISI 316LVM stainless steel, we calculated the DCF amounts that would be incorporated on an artificial hip if it were to be completely covered in the proposed coating. Table 3 shows the calculated values, whereas a standard sized artificial hip was used as the input data for the calculation according to the lowest and highest reported sizes of the respective components of the hip, as reported in^98^,^99^. To calculate the surface area of the artificial hip, a simplified calculation was used, namely a combination of two half-spheres (the inside and outside part of the femoral head) and a cylinder-based simplified surface area for the neck part according to^100^. Three options are presented, where the first and second column show the calculated values if only the head or neck were to be covered in the proposed coating, respectively. The third column shows the calculated values in the case of a fully covered artificial hip. To put these values in the perspective of actual treatment with DCF, the lowest and highest safe daily doses for systemic administration of DCF for pain reduction or anti-inflammatory treatment are reported at the bottom of Table 3. The values in brackets besides these values show the DCF amounts, taking into account DCF bioavailability (BU – the fraction of an administered dose of unchanged drug that reaches systemic circulation, 55%) after *per os* (through the mouth) administration^101^. The released amounts from our respective samples were calculated based on the amount of DCF released in the 15 mL Franz diffusion cell after the reported time frame of the measurements.

A comparison of amounts of DCF released from our samples with actual treatments reveals that for the cases with the lowest administered DCF doses, i.e. general pain (27.5 mg after accounting for BU), we can achieve almost half of that dose with the 3CHI3DCF-coated sample, when considering release from the whole artificial hip. In our opinion, this is already an encouraging result, since we can possibly further increase the incorporated DCF amount by adding additional layers on top of the as-prepared sample.

### Biocompatibility testing

The biocompatibility of materials intended for biomedical applications is always of utter importance^102^,^103^. The main objective of our biocompatibility testing was therefore to assess if the proposed coatings release any toxic degradation products that could hinder cell growth, to evaluate possible local DCF overdoses that could also potentially harm the growing osteoblast cells, and finally to assess the viability of the osteoblast cell culture directly on the as-prepared samples. The second objective was to determine if the proposed coating composition has any measurable effect on the growth of the osteoblast cells, when compared to the control measurement (ADMEM + 5 wt.% FBS). For the control measurement, pure growth media was used for comparison in the case of measurement on the withdrawn samples during *in vitro* drug release, and glass slides in the case of testing directly on the samples. A positive result means potentially better osteointegration, hence a promising direction for future testing and development.

**Figure 9:**
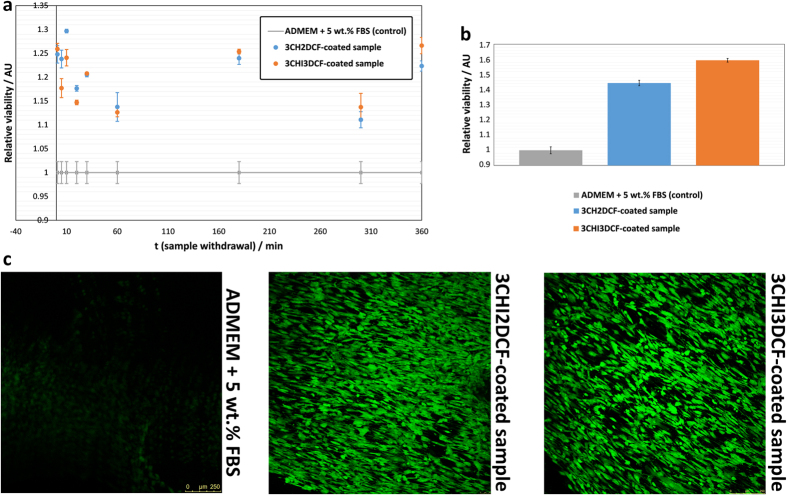
Biocompatibility testing results: (**a**) the results are shown as obtained based on using withdrawn samples during *in vitro* release testing after the desired time intervals, (**b**) results based on the evaluation of biocompatibility directly on the AISI 316LVM stainless steel substrates, and (**c**) confocal microscopy images obtained after staining of the sample and control using the Live/Dead assay.

All tests were performed on our own osteoblast cell culture, which was isolated from a patient after written consent was given as described in the materials and methods section (Fig. 9). In comparison with other available studies, which are mostly conducted on either animal-derived or cancer-transformed osteoblast-like cells^104^,^105^,^106^, using primary human-derived cells has many advantages. The most important among them is the possibility to obtain results-based conclusions with regard to an actual clinical setting. Using purchased cells, on the other hand, probably means a more controlled experiment, since the cells come with a certificate that guarantees certain properties. However, if we would like to cure an actual patient someday, isolated, primary cells are certainly preferable^107^.

Figure 9a,b shows the combined results obtained from biocompatibility testing on the withdrawn solutions and directly on the substrates. The results from the viability assessment using the Live/Dead assay, are shown in Fig. 9c. An MTT-based assay was used to evaluate cell viability after exposure to the materials we propose. In order to obtain as much information as possible with regard to the possible influence of our materials on osteoblast cells during their application, the actual situation was simulated by releasing the DCF amount with possible CHI degradation products after desired time intervals. The withdrawn samples during the *in vitro* drug release testing were pipetted together with a chosen amount of cells into plastic containers and left until confluence was reached. In order to assess the actual influence of the materials we propose, control samples (ADMEM + 5 wt.% FBS) were prepared, using only the growth media and cells. The results are as shown in Fig. 9 for consecutive times that correspond to the withdrawn samples as described above in the discussion of the release results.

We can immediately observe that regardless of the time, samples 3CHI2DCF-coated and 3CHI3DCF-coated outperform the control sample. This means that regardless of the DCF and CHI concentration, which increases with time up to 360 minutes (since at that time the maximum concentration of DCF was measured, these are also the final samples in our testing), all samples are not only biocompatible, but in most cases even an increase in the number of osteoblast cells grown in the same period of time is observed.

This result is important in two regards. Firstly, the as-prepared samples are biocompatible regardless of DCF concentration, which is the highest after 360 minutes, and regardless of possible CHI degradation products that could occur during the same period of time. CHI has already (as mentioned above) been proven to be biocompatible^87^,^88^,^108^. And secondly, these results are very promising with regard to our desire to prepare coatings on AISI 316LVM stainless steel that would promote osteointegration.

To get additional insight into the cell viability of the osteoblast cell culture with our samples, an additional MTT assay was performed directly on the as-prepared samples. Again, we can observe a much improved cell viability, when compared to the control samples (in this case glass slides). These results are shown in Fig. 9b. These results are in complete agreement with the statements above, and again confirm the high potential of the developed coatings for further assessment in relation to bone tissue engineering applications.

Finally, a Live/Dead assay was performed in order to show the cell viability also by visual means directly on the substrates (Fig. 9c). No dead cells were observed on both samples. Furthermore, a much higher intensity of the green light (as observed by confocal microscopy) is in agreement with the previous findings, namely that both samples by far outperform the control samples.

Extensive additional testing is required in order to evaluate the actual mechanism behind the promotion of cell proliferation, but this is outside the scope of this study. Nevertheless, based on the obtained results, we are quite positive that our proposed preparation strategy has great potential with regard to possible future application in orthopedic procedures.

## Conclusion

In this study novel bioactive layer-by-layer coatings on AISI 316LVM stainless steel using the non-steroid anti-inflammatory drug diclofenac (DCF) and polysaccharide chitosan (CHI) are presented. The most important evaluated characteristics of such materials are their corrosion resistance and biocompatibility. Based on this, we first examined the corrosion susceptibility of coated AISI 316LVM steel by employing electrochemical impedance spectroscopy and cyclic polarization measurements. Next, surface morphology was examined by 3D-profilometry and AFM techniques. Time-dependent DCF release from these coatings was also tested. Finally, biocompatibility and osteointegration studies were performed. The main findings are the following:

- The corrosion of AISI 316LVM stainless steel in physiological solution is under mixed kinetic-controlled and diffusion-controlled processes.
- CHI-based and DCF-based coatings do not accelerate the corrosion rate of AISI 316LVM stainless steel. In fact, these two coatings even provide a certain degree of corrosion protection compared with uncoated AISI 316LVM stainless steel. The slowest localized initiation rate occurs for the CHI-coated sample (the 3CHI2DCF-coated sample), followed by uncoated and 3CHI3DCF-coated samples. The slowest crevice corrosion rate occurs for the 3CHI3DCF-coated sample. However, the differences between these three samples are not large.
- The as-prepared coatings enable a very fast initial release (burst) in the first 30 minutes, which is followed by fast release maintained until 90% of the DCF is released in the first 6 h. From 6 h on, a release plateau is maintained, until all of the drug is released after 24 h.
- Such release is very interesting for orthopedic applications (e.g. hip replacements), where the patient wakes up in pain. Until systemic drug application is possible in an efficient and safe manner, such approach could also prevent possible initial inflammation due to the implant.
- The biocompatibility of the presented coatings was proven on primary human-derived osteoblasts.
- The results on osteoblasts entail a possible improvement in osteointegration if the presented coatings are applied.

## Additional Information

**How to cite this article**: Finšgar, M. *et al*. Novel chitosan/diclofenac coatings on medical grade stainless steel for hip replacement applications. *Sci. Rep.* **6**, 26653; doi: 10.1038/srep26653 (2016).

## Figures and Tables

**Table 1:** The composition of AISI 316LVM in wt.% as specified by the supplier.

Cr	Ni	Mo	C	Si	Mn	P	S	N	Cu	Fe
17.53–18.90	14.37–14.94	2.40–2.82	0.011–0.023	0.19–0.57	1.15–1.52	0.018–0.025	0.001–0.009	0.05–0.08	0.03–0.14	balance

**Table 2:** Fitted EIS parameters using the EEC model in Fig. 5 for uncoated and 3CHI3DCF- or 3CHI2DCF-coated AISI 316LVM stainless steel in physiological solution after 1 h, 3 h, 5 h, 7 h, and 10 h of immersion.

Immersion time [h]	χ^2^	*R*_Ω_	*Q*_1_	*n*_1_	*R*_1_	*C*_1_	*Q*_2_	*n*_2_	*R*_2_	*C*_2_	*W*
Uncoated sample											
1	0.69	25.08	41.11	0.92	28.46	40.21	98.28	1.00	262.06	98.28	21.03
3	0.05	25.93	36.13	0.92	11.19	31.96	54.68	0.99	584.46	56.24	15.01
5	0.03	26.37	34.48	0.92	13.57	30.97	44.19	0.97	861.78	50.69	14.40
7	0.02	26.60	33.34	0.92	14.12	29.67	40.46	0.95	1116.14	49.53	13.68
10	0.02	26.82	32.13	0.92	15.04	28.68	38.26	0.94	1461.80	48.80	12.93
3CHI3DCF-coated sample											
1	0.68	24.96	50.57	0.91	51.86	55.80	92.29	1.00	245.00	92.29	15.66
3	0.23	25.63	44.18	0.91	95.68	50.73	93.10	0.99	519.00	93.77	9.25
5	0.03	26.05	41.31	0.91	98.68	47.23	110.23	0.93	788.55	116.67	7.79
7	0.02	26.25	39.56	0.92	109.79	45.44	88.85	0.93	973.90	96.34	7.26
10	0.03	26.53	37.52	0.92	122.77	43.18	109.03	0.79	1475.50	840.05	7.17
3CHI2DCF-coated sample											
1	0.52	25.14	41.31	0.89	23.49	40.54	71.00	1.00	351.30	71.00	15.60
3	0.22	25.96	34.51	0.89	28.50	34.11	56.45	1.00	716.40	56.45	10.89
5	0.23	26.42	32.31	0.89	30.85	32.10	54.81	0.93	1107.50	67.03	9.28
7	0.01	26.61	31.68	0.89	33.67	31.97	47.28	0.86	1370.00	70.62	11.10
10	0.26	26.89	29.69	0.89	47.29	30.83	42.12	0.74	2277.50	180.30	10.73

Units: χ^2^ [×10^−3^], *R*_Ω_ [Ω cm^2^], *R*_1_ and *R*_2_ [kΩ cm^2^], *Q*_1_ and *Q*_2_ [μΩ^−1^ cm^−2^ s^n^], *W* [μΩ^−1^ cm^−2^ s^1/2^]; *C* [μF cm^−2^] was calculated as *C*_x_ = (*R*_x_*Q*_x_)*n*_x_/*R*_x_.

**Table 3:** Calculated DCF amounts on a hypothetical artificial hip.

	Head only	Neck only	Whole artificial hip
Artificial hip surface area (based on REF) (mm^2^)	13,000–23,000	17,000–27,000	30,000–50,000
Calculated possible amount of DCF released with the same composition as in the 3CHI2DCF-coated sample (mg)	3.2–5.6	4.0–6.6	7.2–12.2
Calculated possible amount of DCF released with the same composition as in the 3CHI3DCF-coated sample (mg)	3.6–6.4	4.6–7.4	8.2–13.8
SAFE DAILY DCF DOSES			
Treatment	Safe daily dose (mg)	Safe daily dose based on BU of 55% (mg)	
Dysmenorrhea/migraine/general pain treatment (lowest daily doses)^101^	50	27.5	
Rheumatoid arthritis treatment (highest daily doses)^101^	225	123.75	

Average male and female daily doses of DCF are presented at the bottom of the table for comparison^101^. The surface area of the AISI 316LVM stainless steel substrate is 706.9 mm^2^.
